# Osmotic/ionic status of body fluids in the euryhaline cephalopod suggest possible parallel evolution of osmoregulation

**DOI:** 10.1038/srep14469

**Published:** 2015-09-25

**Authors:** Tatsuya Sakamoto, Satoshi Ogawa, Yudai Nishiyama, Chiaki Akada, Hideya Takahashi, Taro Watanabe, Hiroyuki Minakata, Hirotaka Sakamoto

**Affiliations:** 1 Ushimado Marine Institute, Faculty of Science, Okayama University, Setouchi, Japan; 2 Atmosphere and Ocean Research Institute, University of Tokyo, Kashiwa, Japan; 3 Suntory Foundation for Life Sciences, Mishima, Osaka, Japan

## Abstract

Acclimation from marine to dilute environments constitutes among the dramatic evolutionary transitions in the history of life. Such adaptations have evolved in multiple lineages, but studies of the blood/hemolymph homeostasis mechanisms are limited to those using evolutionarily advanced Deuterostome (chordates) and Ecdysozoa (crustaceans). Here, we examined hemolymph homeostasis in the advanced Lophotrochozoa/mollusc, the other unexplored taxa, and its possible regulation by the vasopressin/oxytocin superfamily peptides known to be implicated in fluid homeostasis in Chordata and Arthropoda. The hemolymph osmotic and ionic status in the euryhaline cephalopod (*Octopus ocellatus*) following transfer from 30-ppt normal seawater to 20 ppt salinity indicate hyperosmo- and hyperionoregulatory abilities for more than 1 week, as in crustaceans and teleost fish. While ventilation frequency decreased by 1 day, Na^+^/K^+^-ATPase activity, which has been generally implicated in ion transport, was induced in two of the eight posterior gills after 1 week. In addition, the octopuses were intravenously injected with 1 or 100 ng/g octopressin or cephalotocin, which are *Octopus* vasopressin/oxytocin orthologs. After 1 day, octopressin, but not cephalotocin, decreased the hemolymph osmolality and Ca concentrations, as well as urinary Na concentrations. These data provide evidence for possible parallel evolution in hyperionoregulatory mechanisms and coordination by conserved peptides.

Acclimation from a marine to a dilute environment constitutes among the most dramatic evolutionary transitions in the history of life, but a number of taxa have succeeded^1^,^2^,^3^. Most animals evolved in the sea and marine invertebrates tend to possess body fluids with an ionic composition that resembles the surrounding seawater. Adapting to dilute environments can result in large changes in the osmotic concentration of hemolymph for osmoconforming marine invertebrates, necessitating the isosmotic regulatory responses in the cells of these animals^4^,^5^,^6^. On the other hand, this also poses great challenges in maintaining essential ions against concentration gradients^7^,^8^. Since organisms in very dilute environments must maintain elevated hemolymph/blood concentrations relative to the environment, evolution of hemolymph/blood regulation constitutes a critical step in adapting to such habitats.

In contrast to decapod crustaceans and teleost fish, little is currently known about hemolymph regulation in molluscs, although the evolutionary competition of fish and molluscs is believed to draw on a variety of physiological functions in cephalopod molluscs^9^, such as a high degree of mobility, high metabolic rates, well-developed neuronal systems and efficient sensory organs^10^,^11^,^12^,^13^. Some stenohaline cephalopod species are weak hypoosmoregulators or osmoconformers, with hemolymph that is slightly hypoosmotic or isoosmotic with respect to the environmental seawater^14^,^15^. This is probably closely related to their comparatively thin (and ion-permeable) skin, which serves as a respiratory organ^16^. In estuarine species, however, osmotic and ionic regulation must be particularly responsive. *Octopus ocellatus* is a common euryhaline cephalopod with a habitat of estuaries in eastern Asia that have high freshwater input during the long spell of rainy weather in early summer and in typhoon events. This species can adapt to significant dilution of the habitat by rainfall and runoff, and survive at least for several days^17^. Among cephalopods, octopods have much larger volumes of renal sacs, including renal appendages, than decapods. In octopods, most excretion occurs in renal appendages with a complex external surface^13^,^18^.

In the *Octopus* species, two peptides of the vasopressin (VP)/oxytocin (OT) superfamily, octopressin (OP) and cephalotocin (CT), have been identified in *Octopus vulgaris*^19^,^20^, as the first demonstration of the co-occurrence of these two peptides in an invertebrate species. In the evolution of molluscs, cephalopods seem to have obtained these two molecules, similarly to jawed vertebrates^21^. The peripheral actions of VP and OT and their orthologs is established in vertebrates: members of the VP family are typically involved in body fluid regulation (e.g., water absorption as an antidiuretic hormone in higher vertebrates), and those of the OT family stimulate uterine smooth muscle contraction during labor and milk ejection from mammary glands during lactation. In invertebrate species, peptides of the VP/OT superfamily seem to be involved in similar processes to those described in vertebrates^22^,^23^,^24^,^25^,^26^,^27^,^28^, but there are no data regarding their roles in mollusc osmotic/ionic balance.

In this context, *O. ocellatus* is an appropriate species in which to test the hypothesis that euryhaline molluscs have osmotic/ionic regulation controlled by VP/OT orthologs. In this study, we examined the influence of a decrease in ambient salinity on the osmotic and ionic concentrations in hemolymph and urine, as well as on the activity of Na^+^/K^+^-ATPase, a central player in osmotic and ionic regulation, in the gill and renal tissues. We then examined the effects of OP and CT on these parameters, and our results show possible parallel evolution in the hyperionoregulatory mechanisms.

## Results

*O. ocellatus* were maintained in tanks supplied with a flow of 30-ppt seawater (SW).

### Effects of reduced salinity

#### Ventilation rate

**Figure 1:**
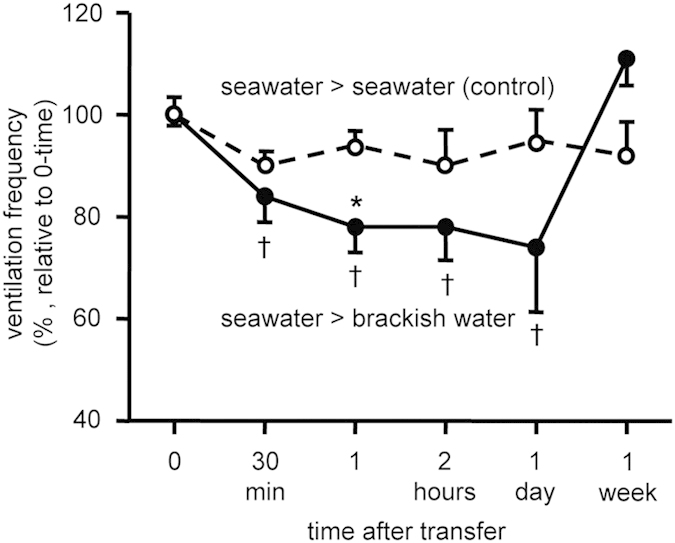
Ventilation responses of the octopus *O. ocellatus* exposed to 20-ppt brackish water. Values are means ± SEM, n = 4–6 animals. Representative results of two independent experiments are shown. *P < 0.05 vs. 30-ppt seawater control at a given time point; †P < 0.05 vs. zero-time values (44.6 ± 1.3 breaths min^–1^ in 30-ppt seawater).

Videos were used to quantify changes in ventilation rate of octopuses after exposure to brackish water (BW, 20-ppt SW). The mean ventilation rate of 44.6 ± 1.3 breaths min^–1^ before BW exposure decreased to below control rates after 1 day, but returned to the control values after 1 week (Fig. 1).

#### Hemolymph and urine

**Figure 2:**
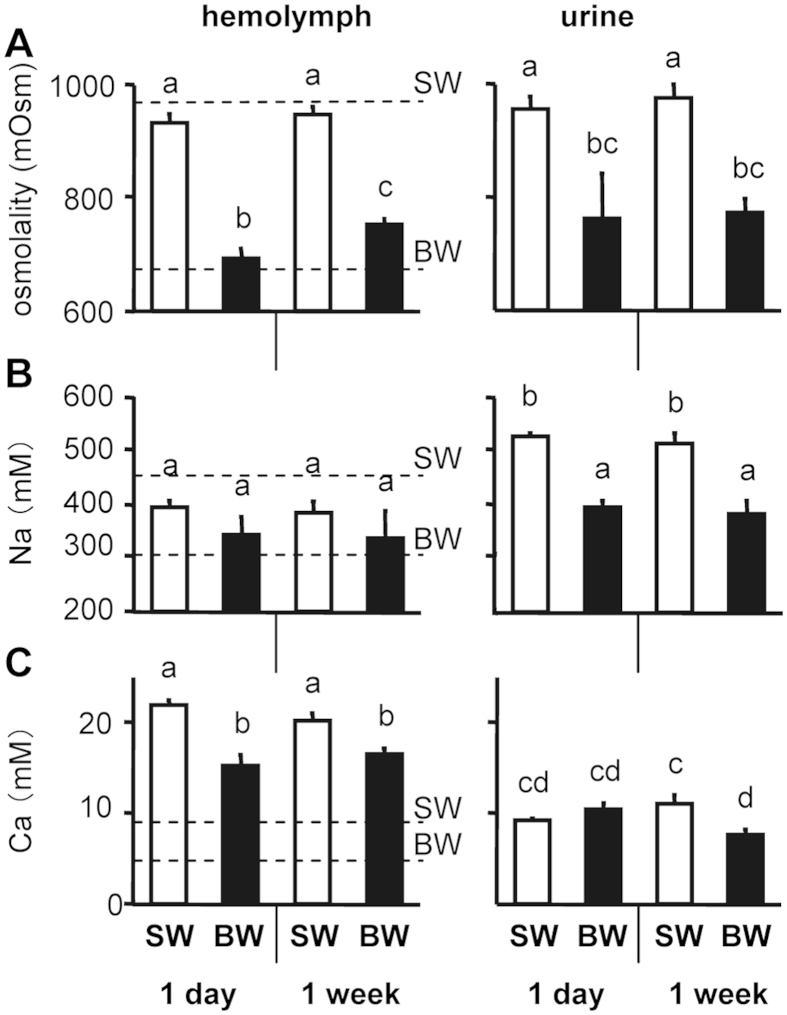
Osmolality and ion concentrations of body fluids of the octopus *O. ocellatus* in brackish water. Osmolality (**A**), Na^+^ (**B**) and Ca (**C**) concentrations of hemolymph and urine of the octopus *O. ocellatus* exposed to 20-ppt brackish water (BW) for 1 day and 1 week. Values are means ± SEM, n = 6–10 animals. Dotted lines indicate osmotic and ionic concentrations of seawater (SW) and BW. Representative results of two independent experiments are shown. Letters denote differences among means (P < 0.05); means with the same letter are not significantly different.

**Figure 3:**
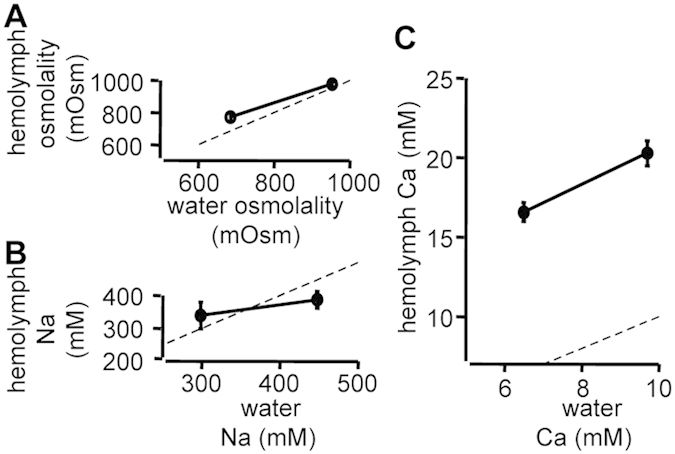
Hemolymph osmolality and ion concentrations of the *O. ocellatus* in relation to the medium values. Osmolality (**A**), Na^+^ (**B**) and Ca (**C**) concentrations in the hemolymph of the octopus *O. ocellatus* acclimated for 1 week in 20- or 30-ppt salinity (mean ± SEM, n = 6 to 10), in relation to the measured values of the medium. Error bars are not shown when smaller than the symbols (**A**). Dotted lines indicate isosmotic and isoionic relationships. Drawn from the data in Fig. 2.

Octopuses exposed to BW for 1 day showed decreases in hemolymph osmolality. This lower osmolality in octopuses in BW was observed for 1 week. However, the osmolality of the hemolymph returned towards the original, control value in 30-ppt SW, and was about 100 mOsm/kg hyperosmotic to that of BW. The osmolality of the urine changed in parallel and was isoosmotic to that of the hemolymph (Fig. 2A). The relationship between acclimated (1 week) octopus hemolymph and water osmolality had a slope of 0.61 (Fig. 3A).

For hemolymph Na concentrations, exposure to BW caused no significant change over 1 week. Na concentrations in urine were higher than those in the hemolymph, and octopuses in BW showed decreases in urinary Na throughout the study, unlike the findings for hemolymph Na (Fig. 2B). The hemolymph Na appeared to be hyporegulated in SW and hyperregulated in BW, above and below the isoionic point, respectively. The relationship between hemolymph and water Na had a slope of 0.35 (Fig. 3B).

Hemolymph Ca concentrations were higher than the medium Ca^2+^ concentration at both salinities over the experimental period. After exposure to BW, hemolymph Ca decreased after 1 day and remained low throughout the experiment (Fig. 2C). The relationship between hemolymph and water Ca had a slope of 0.68 (Fig. 3C). Lower urinary Ca concentrations were always observed relative to those in hemolymph. After 1 week, octopuses exposed to BW also showed reduced urinary Ca concentrations compared to controls (Fig. 2C). Considerable portions of Ca in the hemolymph of cephalopods is probably complexed (protein bound), unlike other ions^14^, and free (unbound) Ca, which is more relevant, could not be measured by ion chromatography (AV10, Shimadzu, Kyoto, Japan) using a cation-exchange column (IC-C3, Shimadzu, Kyoto, Japan), possibly due to the high levels of Cu^2+^, Zn^2+^ and amines (unpublished observation).

#### Na^+^/K^+^-ATPase activity

**Figure 4:**
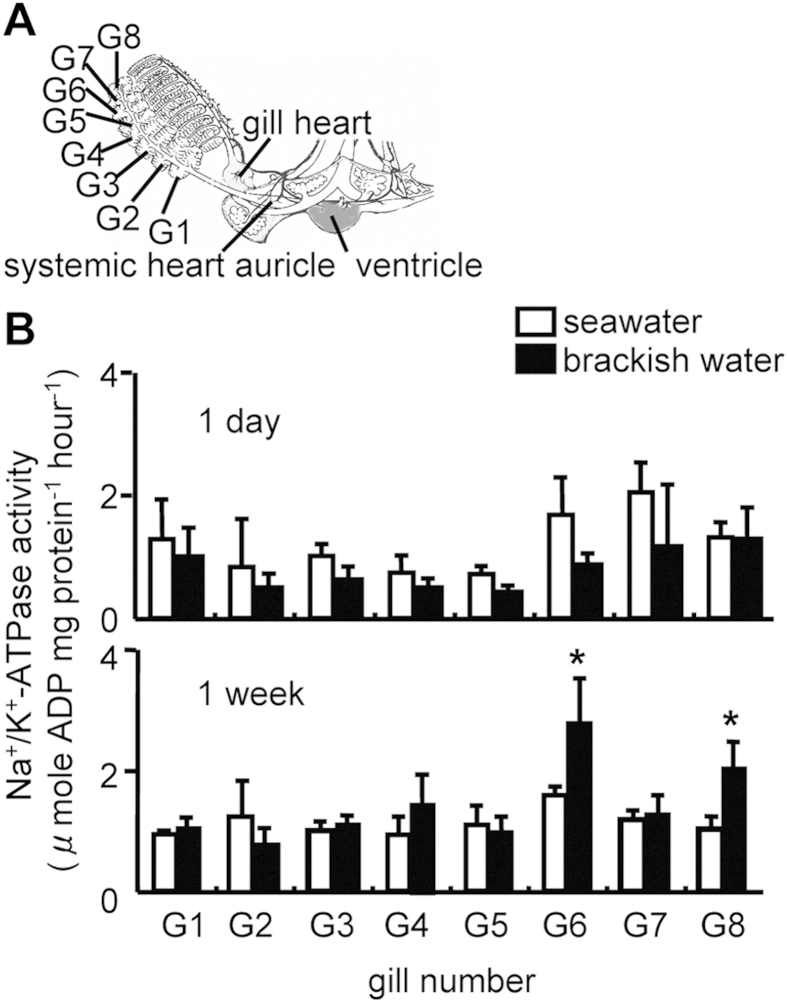
Na^+^/K^+^-ATPase activity in individual gills of the octopus *O. ocellatus* exposed to brackish water. Octopus gills shown on the right side only (**A**). Dissection from the ventral surface; the dorsal aorta running forward from the systemic heart is concealed in this view. Na^+^/K^+^-ATPase activity in individual gills of the octopus *O. ocellatus* exposed to 20-ppt brackish water for 1 day and 1 week (**B**). Values are means ± SEM, n = 6–10 animals. Representative results of two independent experiments are shown. *P < 0.05 vs. 30-ppt seawater control at a given time point.

Analysis of Na^+^/K^+^-ATPase activity showed a gill-specific response to dilute salinity. One week after exposure to BW, Na^+^/K^+^-ATPase activity in gills 6 and 8 increased (Fig. 4). This unique response of the activity was observed in population cohorts caught in two consecutive years. Renal Na^+^/K^+^-ATPase activity was unchanged in octopuses exposed to BW compared with that in controls (Supplementary Figure S1 online).

### Effects of OP/CT

**Figure 5:**
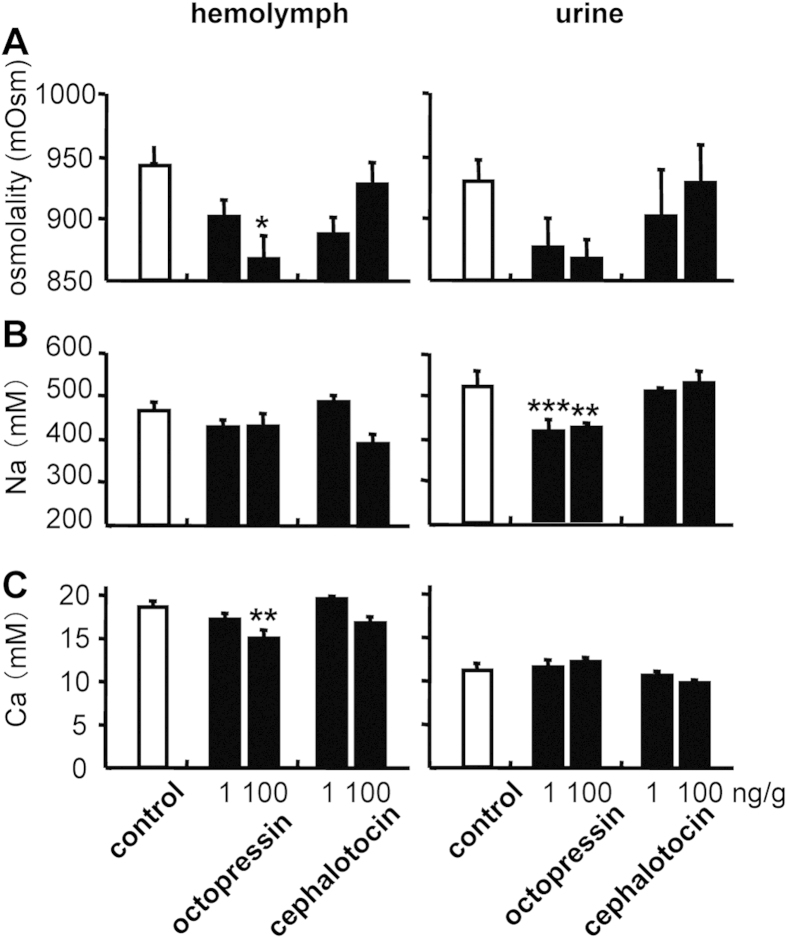
Body fluid osmolality and ion concentrations of the *O. ocellatus* injected with octopressin or cephalotocin. Osmolality (**A**), Na^+^ (**B**) and Ca (**C**) concentrations of hemolymph and urine of the octopus *O. ocellatus* 1 day after injection of octopressin or cephalotocin. One or 100 ng/g of octopressin, cephalotocin or vehicle was injected. Values are means ± SEM, n = 4–8 animals. Significant differences vs. saline-injected controls are indicated: *P < 0.05; **P < 0.01; ***P < 0.001.

Ventilation rates did not differ significantly between control and OP-injected groups (P > 0.05) or between control and CT-injected groups (P > 0.05; Supplementary Figure S2 online). As seen in Fig. 5, OP treatment (100 ng/g) decreased the hemolymph osmolality and Ca concentrations, as well as urinary Na concentrations, after 1 day.

In contrast, OP produced no significant effects on Na^+^/K^+^-ATPase activity in gills 6 or 8 or in the kidney compared to the control group 1 day after the last injection (P > 0.05; Figures S3 and S4). Neither body fluid parameters nor Na^+^/K^+^-ATPase activity differed significantly between the control and CT-injected groups (P > 0.05; Fig. 5 and Supplementary Figures S3 and S4 online). There were no sex differences in the effects of OP or CT, and no other physiological effects of OP or CT were found other than those in fluid metabolism.

## Discussion

A number of taxa have independently been able to breach the formidable barrier of acclimation from a marine to a very dilute environment, whereas most invertebrate species have been unable to adapt^2^. Such acclimation imposes serious challenges for ionic and osmotic regulation, in terms of acquiring ions from dilute solutions against steep concentration gradients. Previous studies have shown these ion uptake activities in crustaceans and teleost fish^29^,^30^. In this study, we show for the first time that such osmo- and ionoregulation can also be found in cephalopods; the osmotic and ionic concentrations in the hemolymph of euryhaline *O. ocellatus* appear to be regulated when the salinity of the environment decreases. In addition, this is also the first study that implies a role of the intrinsic VP/OT-superfamily peptide in body fluid regulation in a mollusc species.

The initial objective of the study was to assess physiological events that occur in *O. ocellatus* over a week at reduced salinity comparable to those in their natural environment of the Bisan Strait^31^. While many studies have looked at salinity stress in decapod crustaceans and teleost fish^29^,^30^, few, if any, have examined the effects in mollusks, except their isosmotic intracellular regulation^4^,^5^,^6^. As soon as the salinity decreased, octopuses became quiescent, with a decreased ventilation rate and closed siphon. Such a closure response has been shown to isolate the mantle cavity (branchial chamber) from the surrounding low salinity water^5^,^32^,^33^. Because of the rapid isolation response and diffusive ion loss into a closed area, the water in the mantle cavity should be held at a higher salinity than the surrounding water. The sealed chamber appears to result in a reduction in oxygen uptake, and these periods of 'breath holding' can only last for a short time before oxygen reserves are depleted, forcing opening and a subsequent increase in oxygen uptake^34^. It has been suggested that organisms respond to a dilution of the medium by exhibiting an increase, decrease or no change in respiration levels^35^, with euryhaline organisms showing an increase and stenohaline organisms exhibiting a decrease in respiratory parameters, but this has been difficult to substantiate^24^,^36^. The results from the present study suggest that *O. ocellatus* may prioritize the counteraction of passive ion leakage shortly after exposure to low salinity.

Slopes resulting from regression analysis between hemolymph osmotic/ionic concentrations and those in medium at given salinity have been used to determine the ability of aquatic organisms to osmo- and ionoregulate^4^,^5^,^37^,^38^. The closer the deviation of slope to the isosmotic and isoionic line (slope = 1), the lower the ability of the organisms to osmo- and ionoregulate. Osmotic or ionic status of body fluids has been investigated in very few cephalopods^14^,^15^ and this study using *O. ocellatus* is the first to show a hyperosmoregulatory ability (Figs 2A and 3A). Importantly, the hemolymph Na concentration appeared to be hyporegulated at 30 ppt and hyperregulated at 20 ppt (Figs 2B and 3B). As reported for some stenohaline octopuses^14^,^15^, the urine is virtually isosmotic to the hemolymph, but the urine Na^+^ levels were higher than those in the hemolymph, which may play a role in maintaining Na^+^ balance between the hemolymph and higher-salinity (30 ppt) external media. On the other hand, like several freshwater crabs (e.g., potamoid crabs in general), the hyperregulating *O. ocellatus* at 20 ppt could not produce urine hypoosmotic to the hemolymph, although various crayfish species produce copious, dilute urine (10–20% that of the hemolymph concentration), thus conserving salts but excreting their osmotic water load, as also established in teleost fish acclimated to ion-poor environments^30^. As an alternative strategy, *O. ocellatus* at 20 ppt may produce an increased flow of urine to counterbalance the osmotic water load, but the urine volume in the small cephalopod could not be measured^14^. More clearly, the *O. ocellatus* appears to absorb Na^+^ from dilute seawater via the branchial Na^+^/K^+^-ATPase, an ion transporter that has been studied intensively in osmoregulating aquatic organisms^39^. In this study, we observed significant increases in the Na^+^/K^+^-ATPase activity of gills 6 and 8 one week after acclimation to 20-ppt salinity (Fig. 4), similarly to the posterior gills in many crustaceans structurally and functionally specialized as the principal site of osmoregulatory ion transport^30^,^37^,^38^,^40^. The involvement of other transport proteins and transport-related enzymes (e.g., a Na^+^/H^+^ antiporter, carbonic anhydrase, Cl^−^/HCO3^−^ exchanger, Na^+^/K^+^/2Cl^−^ cotransporters, and V-ATPases) and/or the acid-base balance/ammonium transport cannot be excluded^41^, but little is known about the related parameters during acclimation to different salinities. Regarding Ca^2+^ changes (Figs 2C and 3C), the regulation was unclear because we could not measure the relevant free moiety, but the decreased urine Ca following the exposure to BW suggests reabsorption of Ca^2+^ from urine. As in crustaceans^37^,^38^,^42^, hemolymph complexed calcium may serve as an internal reserve for maintaining free Ca^2+^ levels in the hemolymph. In addition to the Ca^2+^ channel, Ca^2+^-ATPase and Na^+^/Ca^2+^ exchanger, the potential energy of the Na^+^ gradient established by the branchial Na^+^/K^+^-ATPase activity may also affect transport of Ca^2+^ in hemolymph.

After injection of OP, *O. ocellatus* showed decreases in hemolymph osmolality and Ca, as well as in urinary Na^+^, whereas an injection of CT did not have these effects. Roles of OP/CT orthologs in cephalopod memory processes and reproductive physiology (contractile activity of the organ responsible of gamete emission) have been reported^20^,^43^,^44^, but the present study is the first in a mollusc species to demonstrate the physiological functions of intrinsic VP/OT-related peptides in body fluid regulation, which is well-established in mammals and has also been described through the phylum Chordata and in Arthropoda^24^,^25^,^28^,^45^. These peptides also modulate reproduction in some phyla, suggesting that generalizations about the relevant functions cannot be made until experiments are conducted in larger samples of taxa^28^,^46^.

The mechanisms by which OP affects the hemolymph and urine cannot be completely understood at present. The distribution of a receptor for OP in *Octopus* tissues such as nervous tissues, the gastrointestinal tract and circulatory systems, including gills^21^, may explain these effects, but no significant influence of OP on Na^+^/K^+^-ATPase activity in the gills or kidney was observed. OP might be involved in controlling aquaporins and other transporters described above for selective secretion and (re)absorption of ions in these organs. This peptide may regulate smooth muscle contractions for peristalsis and production of hydrostatic pressure for urine formation in the kidney. OP, rather than CT, is involved in contractile activity of visceral and smooth muscles in *Octopus* species *in vitro*, while neither circulating OP nor its renal receptor was detected^20^,^21^,^25^,^44^. As demonstrated in vertebrates^47^,^48^, therefore, these peptides may also act within the confines of the CNS. OP and its receptor mRNA are distributed to a large degree in the *Octopus* CNS^21^, suggesting central roles of this peptide, including those associated with body fluid regulation. Sex differences in the expression of OP receptor in these organs have not been reported, unlike VP receptors in mammals, whereas OP receptor mRNAs are widely distributed in tissues associated with female reproduction, but not in testis^21^.

In summary, we showed in this study that reduced environmental salinity elicits changes in ventilation rate, as well as those in osmolality and ionic concentrations in hemolymph and urine of *O. ocellatus*, occurring mostly in the first 24 h. We also found increased Na^+^/K^+^-ATPase activity in specific gills after 1 week. The immediate decrease in ventilation probably plays a role in isolating the gills from the surrounding water and counteracting changes in hemolymph solute concentrations when faced with environmental salinity stress. The branchial Na^+^/K^+^-ATPase activity shows delayed increases in response to low salinity, reflecting activated ion absorption, and these increases are possibly indicative of cellular differentiation associated with the hyposalinity stress response. These hyperionoregulatory responses found here are observed in some phylogenetically diverse taxa entering hyposalinity environments from seawater, but these taxa appear to be evolutionarily advanced (chordates, arthropods and cephalopods among Deuterostome, Ecdysozoa and Lophotrochozoa, respectively) in fluctuating salinities of brackish water^29^,^30^. Thus, this might indicate a possible evolutionary parallelism that is generally relevant to physiological salinity transitions of many species. The study also provides the first evidence for osmo/ionoregulatory effects of the VP/OT-related peptide in a mollusc species, which also suggests that common regulation of body fluid, especially of urine, may have evolved, at least in some phylogenetically taxa with advanced osmo/ionoregulatory ability. To acquire a clearer picture of the physiological characteristics associated with body fluid regulation in cephalopods, future investigations will focus on using molecular tools to examine the expression patterns of genes/proteins of the transporters and the possible ionocytes^49^.

## Methods

### Animals

*O. ocellatus* adults (weight 50 to 120 g) were collected from Bisan Strait, into which the Yoshii River flows (34°40′ N:134° E). Experiments were performed at the nearby Ushimado Marine Institute of Okayama University. The animals were maintained in aerated tanks under a natural light regime and supplied with a continuous flow of 30-ppt SW (448 mM Na^+^, 506 mM Cl^−^, 9.7 mM Ca^2+^, 9.7 mM K^+^, 994 mOsm kg^−1^). Water temperature was 22 ± 2 °C. The octopuses were fed live crabs (*Gaetice depressus*) and clams (*Ruditapes philippinarum*). The octopuses were acclimated to these laboratory conditions for at least 2 weeks before experimentation. All animals were handled, maintained and used in accordance with international standards on animal welfare and the Guidelines for Animal Experimentation established by Okayama University and accepted internationally, as well as in compliance with national regulations. All animal experimental procedures were approved by the Committee for Animal Research, Okayama University (IACUC). No specific permits were required for the described field studies, which did not involve endangered or protected species. The animals were anesthetized with 2% ethanol before sacrifice and all efforts were made to minimize suffering.

### Experimental protocol

A reduced salinity treatment was designed to simulate conditions of increased fresh-water input that *O. ocellatus* encounter in their natural environment. For example, in the estuary habitats, salinity levels can fall rapidly during heavy rainfall runoff. Therefore, the salinity of some tanks was changed from SW to 20-ppt BW (299 mM Na^+^, 337 mM Cl^−^, 6.5 mM Ca^2+^, 663 mOsm kg^−1^) without disturbing the animals by supplying a BW flow. The levels from 20- to 30-ppt salinity represent the range of ambient salinity conditions found in the Bisan Strait^31^. About 30 min was required for complete replacement, and this group stayed in the BW thereafter. To serve as controls, other animals were placed in separate tanks with replacement from 30-ppt SW to SW. The reduced salinity treatments were also chosen based on the range of salinity tolerance (i.e., minimum salinity) for this species, which was determined empirically in our laboratory. Pilot 10-day LC_50_ bioassays indicated that *O. ocellatus* was intolerant of salinity <15 ppt (unpublished data); hence, salinity of 20 ppt was used in the experiments. Salinity was checked with a refractometer, and osmolality and ion concentrations were later confirmed using a vapor pressure osmometer (Wescor Inc. 5500 calibrated with Wescor 290 mmol/kg and 1000 mmol/kg standards, Logan, UT, USA) and an atomic absorption spectrophotometer (Hitachi Z5300; Tokyo, Japan). The ventilations were counted in 5-min periods before treatment (time zero) and after 0.5, 1 and 2 h, 1 day, and 1 week of exposure (n = 4–6 for each group). After 1 day and 1 week, 8–15 animals from each group were sacrificed by decerebration following collection of hemolymph (>50 μl/octopus) with a needle and syringe through the cephalic vein. Access to the renal sac was gained by cutting the medial septum and inverting the mantle. Urine samples (ca. 1 ml) were quickly collected from the paired renal sacs, also using a needle and syringe. Supernatants of centrifuged hemolymph and urine were kept at −80 °C for later analyses. The eight gills were dissected out from the animals, blotted and placed in ice-cold SEI buffer (250 mM sucrose, 10 mM di-sodium EDTA, 50 mM imidazole, pH 7.3), and frozen immediately at −80 °C. Renal tissues were cut away from the renal vein, also immersed in the SEI buffer and frozen. Exposures were replicated twice.

To examine the effects of OP/CT treatment on ventilation rates and on hemolymph and urine parameters, octopuses in SW were injected with 1 or 100 ng/g body weight of synthetic *O. vulgaris* OP (H-Cys-Phe-Trp-Thr-Ser-Cys-Pro-Ile-Gly-NH_2_) or CT (H-Cys-Tyr-Phe-Arg-Asn- Cys-Pro-Ile-Gly-NH_2_)^21^ in a volume of 1 μl/g body weight of saline (filtered, isosmotic artificial seawater [ASW; GEX, Osaka Japan]). The *O. vulgaris* peptides were used, since they are highly homologous even to the cuttlefish counterparts, with only one and two different amino acids^44^. The doses were chosen based on the results of our preliminary studies and published reports on effective physiological doses^20^,^21^,^43^,^44^,^50^. The control group received an equal volume of ASW. On the day of use, each peptide aliquot was diluted in the ASW. Solutions were administered through the neck at a depth of 10 mm. Our earlier experiments showed that Evans blue administered into this portion of the cephalic vein diffuses rapidly into the blood stream. Ventilations were counted after 0, 0.5, 1 and 2 h, and 1 day of treatment (n = 4–9 for each group). One day after the injection, 4 to 9 animals from each group were sacrificed following collection of hemolymph, and urine samples were collected. The effects on Na^+^/K^+^-ATPase activities were examined after injections every second day with 100 ng of OP/g body weight for a total of three injections. This regimen was based on our preliminary analysis of Na^+^/K^+^-ATPase activities showing no significant effects after 1 day, on these activities during salinity acclimation, and on results indicating that 100-ng OP/g had an influence on the hemolymph and urine parameters (see Results). Eight animals from each group were sampled 1 day after the last injection.

### Ventilation rate

For a single octopus settled in an experimental tank, video-taped ventilations were counted for 3 min at each time interval^51^. Changes in VR are shown as a percent of the average ventilation rate in the 5-min period before treatment (set as time zero).

### Osmolality and ionic concentrations in hemolymph and urine

Osmolality determinations were made on 5-μl samples (diluted 1:1 with deionized water) using the vapor pressure osmometer. Na (1 μl sample) and Ca (20 μl sample) were determined after dilution (1:5000 and 1:100) using an atomic absorption spectrophotometer.

### Na^+^/K^+^-ATPase activities in gill and renal tissues

The Na^+^/K^+^-ATPase activity was determined with a linked pyruvate kinase/lactate dehydrogenase-NADH assay^52^, using a method validated for euryhaline invertebrates^37^,^38^. Tissue was homogenized in ice-cold 0.1% deoxycholate SEI buffer (1:9 *w*/*v*) and centrifuged at 5,000 × *g*. The resulting supernatant was diluted and assayed for Na^+^/K^+^-ATPase activity. Each sample of homogenate was plated in quadruplicate samples of 10 μl, with two containing 2.8 mM ouabain. Salt solution (50 μl; 50 mM imidazole, 189 mM NaCl, 10.5 mM MgCl_2_, and 42 mM KCl) and 150 μl of assay mixture (50 mM imidazole, 2 mM phosphoenolpyruvate, 0.16 mM nicotinamide adenine dinucleotide, 0.5 mM adenosine triphosphate, 3.3 U/ml lactic dehydrogenase, and 3.6 U/ml pyruvate kinase) were added to each well. Kinetic data were obtained at a wavelength of 340 nm at 22 °C with a run time of 10 min and intervals of 10 s. The difference between the results with and without ouabain reflects the Na^+^/K^+^-ATPase activity expressed as μmoles ADP per mg protein/h. Total protein in homogenates was measured using a BCA Protein Assay kit (Pierce Chemical Co., Rockford, IL, USA). Assays were run on a microplate reader (Multiskan Ascent, Thermo Electron Corporation, Vaanta, Finland).

For further validation of this system for each tissue, the standard conditions described above were used while varying one factor and keeping all the other parameters constant. Inhibition by ouabain corresponding to the actual measurement of Na^+^/K^+^-ATPase activity, as a function of the ouabain concentration in the reaction mixture, was first examined. Under the standard conditions, the final concentrations of ouabain in the reaction mixture were varied at 0, 0.5, 1.4, 2.8, and 5.0 mM in wells, and maximal inhibition was observed at 2.8 mM ouabain. Thus, this concentration was fixed in examination of other parameters in the remainder of the validation. Optimal conditions for actual analyses of response to reduced salinity and OP/CT treatment were set based on the results. In examinations of the effects of tissue protein concentration on the enzymatic activity in a sample of 0.1 mg protein/10 μl of 0.1% deoxycholate SEI buffer diluted from 1- to 50-fold, activity decreased linearly in proportion to the protein quantity. Therefore, measurements were valid for samples diluted at least twofold, but samples were usually diluted tenfold in this investigation.

### Statistical analysis

Statistics were performed using Statview 4.11 (Abacus Concept). Two- and three-way ANOVA with a repeated measures (time) design (with time within groups [i.e. after application of treatment], and treatment among or between groups, and peptide concentration within groups as factors) followed by the appropriate post hoc test was used to examine differences in ventilation rate between the control and treatment groups. Three-way ANOVA (with treatment among or between groups, and hemolymph vs. urine, and time or peptide concentration within groups as factors) followed by the appropriate post hoc test was used for differences in body fluid parameters. One- and two-way ANOVA were used for differences in Na^+^/K^+^-ATPase activity. All data were checked for normality and equal variances. Where assumptions of normality or equal variances were not satisfied, a nonparametric Kruskal-Wallis tests were used.

## Additional Information

**How to cite this article**: Sakamoto, T. *et al.* Osmotic/ionic status of body fluids in the euryhaline cephalopod suggest possible parallel evolution of osmoregulation. *Sci. Rep.* **5**, 14469; doi: 10.1038/srep14469 (2015).

## Supplementary Material

Supplementary Information
