# Supplementary material for: Osmotic/ionic status of body fluids in the euryhaline cephalopod suggest possible parallel evolution of osmoregulation
Source: Sci Rep. 2015 Sep 25;5:14469. doi: 10.1038/srep14469 (PMC4585917; doi:10.1038/srep14469)
Supplement: Supplementary Information [file srep14469-s1.doc]

**Supplementary Information:**

**Osmotic/ionic status of body fluids in the euryhaline cephalopod suggest parallel evolution of osmoregulation**

Correspondence: ryu@uml.okayama-u.ac.jp

Tatsuya Sakamoto^1^, Satoshi Ogawa^1^, Yudai Nishiyama^1^, Chiaki Akada^1^, Hideya Takahashi^1^, Taro Watanabe^2^, Hiroyuki Minakata^3^, Hirotaka Sakamoto^1^

^1^ Ushimado Marine Institute, Faculty of Science, Okayama University, Setouchi, Japan

^2^ Atmosphere and Ocean Research Institute, University of Tokyo, Kashiwa, Japan

^3^ Suntory Foundation for Life Sciences, Mishima, Osaka, Japan

**Supplementary Information**

**Supplementary Figure S1.** Na^+^/K^+^-ATPase activity in renal tissues of the octopus *O. ocellatus* exposed to 20-ppt brackish water.

**Supplementary Figure S2.** Ventilation responses of the octopus *O. ocellatus* after injection of octopressin or cephalotocin.

**Supplementary Figure S3.** Na^+^/K^+^-ATPase activity in gills 6 and 8 of the octopus *O. ocellatus* treated with octopressin.

**Supplementary Figure S4**. Na^+^/K^+^-ATPase activity in renal tissues of the octopus *O. ocellatus* treated with octopressin.

**Supplementary Figure S1.** Na^+^/K^+^-ATPase activity in renal tissues of the octopus *O. ocellatus* exposed to 20-ppt brackish water for 1 day and 1 week (mean ± s.e.m., n = 6-10 animals). Representative results of two independent experiments are shown. There were no significant differences for each time period (P > 0.05).

**Supplementary Figure S2.** Ventilation responses of the octopus *O. ocellatus* after injection of octopressin or cephalotocin. One or 100 ng/g of octopressin, cephalotocin or vehicle was injected (mean ± s.e.m., n = 4-8 animals). No significant differences were seen (P > 0.05).

**Supplementary Figure S3.** Na^+^/K^+^-ATPase activity in gills 6 and 8 of the control octopus *O. ocellatus* and those treated with octopressin (mean ± s.e.m., n = 8 animals). There were no significant differences for each gill (P > 0.05).

**Supplementary Figure S4.** Na^+^/K^+^-ATPase activity in renal tissues of the control octopus *O. ocellatus* and those treated with octopressin (mean ± s.e.m., n = 8 animals). No significant differences were seen (P > 0.05).
